# Genomic annotation for vaccine target identification and immunoinformatics-guided multi-epitope-based vaccine design against Songling virus through screening its whole genome encoded proteins

**DOI:** 10.3389/fimmu.2023.1284366

**Published:** 2023-11-28

**Authors:** S. Luqman Ali, Awais Ali, Abdulaziz Alamri, Aliya Baiduissenova, Marat Dusmagambetov, Aigul Abduldayeva

**Affiliations:** ^1^ Department of Biochemistry, Abdul Wali Khan University Mardan, Mardan, Pakistan; ^2^ Department of Biochemistry, College of Science, King Saud University, Riyadh, Saudi Arabia; ^3^ Department of Microbiology and Virology, Astana Medical University, Astana, Kazakhstan; ^4^ Preventive Medicine, Astana Medical University, Astana, Kazakhstan

**Keywords:** immunoinformatics, *Songling virus*, reverse vaccinology, molecular docking, MD simulation

## Abstract

*Songling virus* (SGLV), a newly discovered tick-borne orthonairovirus, was recently identified in human spleen tissue. It exhibits cytopathic effects in human hepatoma cells and is associated with clinical symptoms including headache, fever, depression, fatigue, and dizziness, but no treatments or vaccines exist for this pathogenic virus. In the current study, immunoinformatics techniques were employed to identify potential vaccine targets within SGLV by comprehensively analyzing SGLV proteins. Four proteins were chosen based on specific thresholds to identify B-cell and T-cell epitopes, validated through IFN-γ epitopes. Six overlap MHC-I, MHC-II, and B cell epitopes were chosen to design a comprehensive vaccine candidate, ensuring 100% global coverage. These structures were paired with different adjuvants for broader protection against international strains. Vaccine constructions’ 3D models were high-quality and validated by structural analysis. After molecular docking, SGLV-V4 was selected for further research due to its lowest binding energy (-66.26 kcal/mol) and its suitable immunological and physiochemical properties. The vaccine gene is expressed significantly in *E. coli bacteria* through *in silico* cloning. Immunological research and MD simulations supported its molecular stability and robust immune response within the host cell. These findings can potentially be used in designing safer and more effective experimental SGLV-V4 vaccines.

## Graphical Abstract

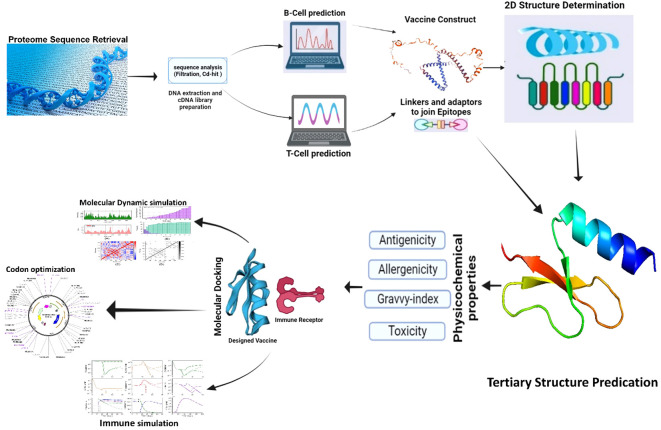

## 1 Introduction

The recent discovered *Songling virus (*SGLV) in China marked a significant moment in pathogenic viruses (1). Its genomic configuration exhibited profound structural homologies with reputable orthonairoviruses, spanning sequence similarities from 46.5% to 65.7%. Phylogenetic analyses situated SGLV distinctly within the Tamdy orthonairovirus group, encapsulating its place within the broader framework of the Nairoviridae family. Microscopic scrutiny validated SGLV’s morphological congruence with the hallmark attributes of orthonairoviruses (2). Notably, it’s essential to mention that SGLV is a single-stranded RNA (ssRNA) virus, contributing to its classification within this viral group.

Functionally, isolated SGLV strains sourced from patients exhibited the capacity to induce prominent cytopathic effects in human hepatoma cells, accentuating its potential for pathogenesis. Between 2017 and 2018, SGLV’s impact on human health materialized, materializing as symptoms encompassing headaches, fever, depression, fatigue, and dizziness. Serological investigations illuminated a pivotal facet: a significant 69% of patients exhibited the development of virus-specific antibody responses during the acute phase (3).

Remarkably, the absence of discernible SGLV viral RNA and the conspicuous scarcity of specific antibodies within healthy cohorts underscored its nuanced selectivity in its interaction with human physiology. Beyond human hosts, SGLV found ecological footing within ticks such as Ixodes crenulatus, Haemaphysalis longicornis, Haemaphysalis concinna, and Ixodes persulcatus within the northeastern precincts of China. Significantly, the viral L segments of SGLV came to the fore, manifesting in 2.2% of spleen samples from great gerbils. BLASTn alignments divulged a compelling narrative of genetic resonance, as the SGLV in great gerbils demonstrated a remarkable alignment of 93.7% (236/252 nucleotides) and 94.0% (78/83 amino acids) with its counterpart detected in human patients with a history of tick encounters within the confines of northeastern China (1). This multifaceted interplay of genetics, ecology, and clinical ramifications has woven a comprehensive tapestry, enriching our comprehension of SGLV’s influence on human health within the geographical landscape of northeastern China (1, 4).

Reverse vaccinology is a cutting-edge strategy that has been widely applied to the introduction of new vaccinations. The strategy aims to combine immunogenomics and immunogenetics with bioinformatics for the development of novel vaccine targets (5). With the recent advancements in genome or protein sequence databases, this rapid *in silico* method has gained significant appeal (6). An innovative vaccine fuses CTL and HTL segments with specialized linkers and adjuvants, showcasing high antigenicity, non-allergenicity, and stability. Molecular docking finds strong binding energy to TLRs, promising robust immunogenic responses. Immune simulation employed to simulates a natural immune response, where the top candidate activates essential immune components (IgG, IgM, T-cells, B-cells, and cytokines), promising protection against the Songling virus (7). Further investigations, including molecular dynamics and computational cloning for efficient E. coli expression, solidify the vaccine’s prospects. Vaccine may recognize and boost immunity against infection in the body. Therefore, predicting allergenicity is a crucial stage in the creation of a neuropeptide vaccine. Immunoinformatics techniques and tools were utilized to design a non-allergic, immunogenic, and thermostable recombinant vaccine against the *Songling virus*, and we expect wet lab researchers to confirm our prediction.

## 2 Methodology

**Figure 1:**
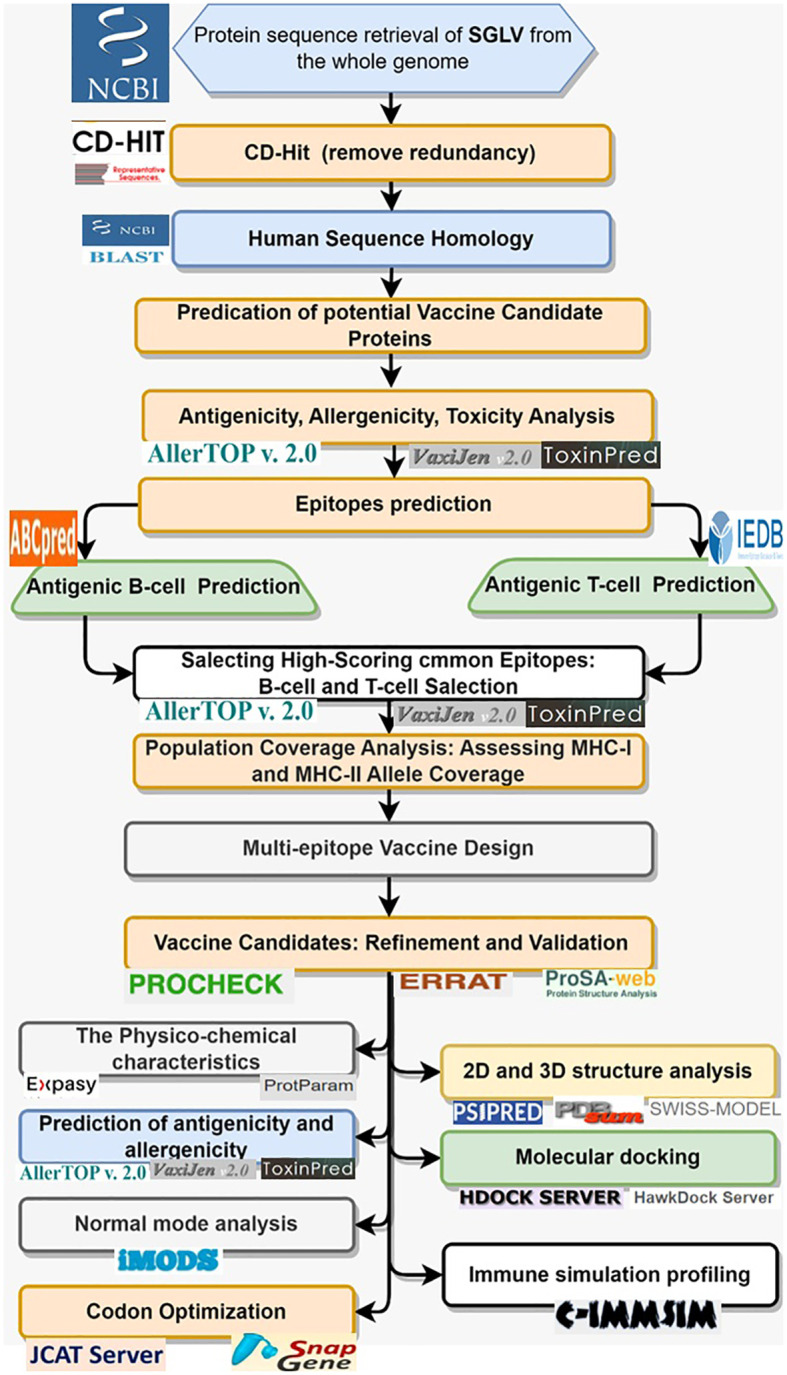
Systematic flow chart of *in-silico* based multi-epitope vaccine design.

The systematic methodology employed in this study to design a multi-epitope vaccine construct targeting the *Songling virus* (SGLV) (**Figure 1**).

### 2.1 Protein sequence retrieval and filtration

*Songling virus* proteome data was downloaded from the National Centre for Biotechnology Information (NCBI) database under reference taxonomy ID: 2795181, and sequences were verified from Virus Pathogen Resource (ViPR) (8, 9). The protein sequence data for SGLV was in FASTA format and submitted to NCBI on May 6, 2023. Different parameters of CD-hit were used for obtaining 85% sequence similarity and removing redundancy to acquire non-paralogous sequences of proteins (10). BLASTp of NCBI was used for sequence homology to human proteins with thresholds of percent identity < 35, query coverage 75, e-value 10-4, bit score <100, and others used as defaults (11). For identification of the allergenicity of SGLV proteins, the AllerTop online server was used (12). The antigenicity was determined by setting the 0.4 parameter in VaxiJen online server (13). For the identification of the toxicity of SGLV proteins, the Toxinpred 3.0 online server was implemented (14).

### 2.2 Prediction of T-cell and B-cell

MHC-I epitopes were predicted using the IEDB MHC-I stickiness predictions program (15) accessed on June 23, 2022. The prediction algorithm used the SMM approach, and sequences were provided in FASTA format. It has been determined that humans will be the host species. The output format was set to XHTML tables, and all other options and parameters were left as default. Similarly, the IEDB MHC-II binding prediction tool (16) accessed on June 25, 2023, was used to predict the MHC-II epitopes by selecting the SMM prediction method. Data was provided in FASTA format. The HLA-DR was chosen as the species/locus couple, and then alleles were chosen using the typical length values associated with each species/locus (17). The other variables were kept at their default settings, and the final result format was set to XHTML table.

The B cell is an important element of the body’s defense system. It is responsible for secreting antibodies that provide long-term immunity (18). For the detection of a continuously growing 12-mer long B-cell lymphoid (BCL) for the selected amino acids with a threshold number of 8.0, ABCPred (http://crdd.osdd.net/raghava/abcpred/) tool was used for this analysis (18). linear B-Cell and MHCI & II overlapped epitopes are selected based on physiochemical properties by employing VaxiJen v2.0, AllerTOP v.2.0, & ToxinPred 3.0 tools (12, 13, 19).

### 2.3 Population coverage

The IEDB population coverage assessment tool (https://tools.iedb.org/population/) was utilized to check the designed vaccine had successfully covered the entire world population (20). Populations research in China, eastern the People’s Republic, southern the Caribbean, the Southeast Asian region, and the ocean was conducted to understand the global nature of the *Songling virus* pandemic. Default values were used to evaluate coverage for MHC class I and class II HLA binding alleles (15). This strategy takes advantage of the worldwide distribution of HLA-binding genotypes to calculate the abundance of certain epitopes.

### 2.4 Multi-epitope vaccine construct design

**Figure 2:**
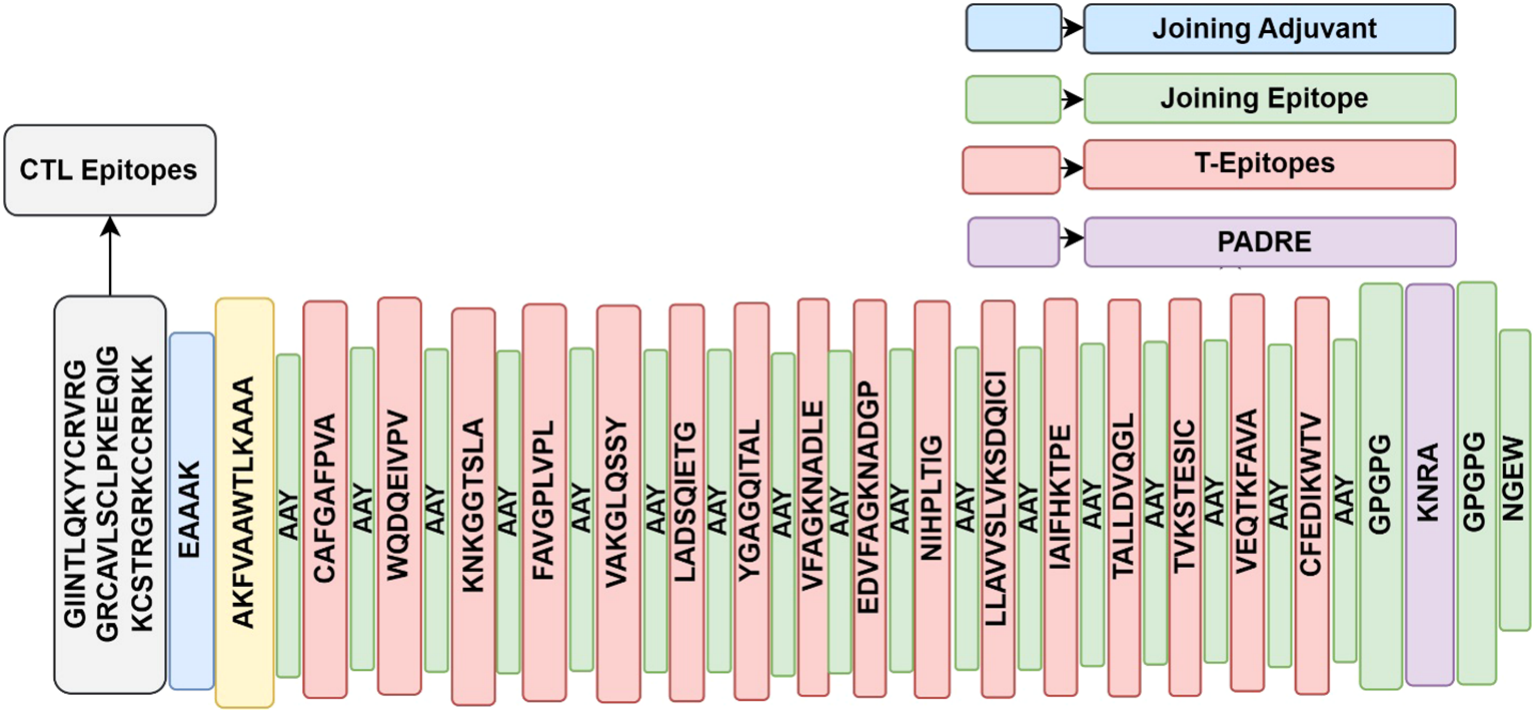
Graphical illustration of multi-epitope vaccine construct, showing CTL, and T-cell epitopes joined by appropriate links and Adjuvant.

Effective vaccine construct design and proper epitope separation depend on all candidate epitopes being connected together through linkers and adjuvants. The B-cell epitope was linked to the CTL targets using the EAAAK, AAY linkers, and the HTL epitopes were linked to the CTL targets using the GPGPG linker (**Figure 2**). To facilitate future glycosylation with a carrier protein, a cysteine residue was included in the N-terminal of the multi-epitope vaccine construct (21). The antigenicity, allergenicity, toxicities, and physicochemical features of the vaccine construct were analyzed further using the ProtParam tool (https://web.expasy.org/protparam/) (19).

### 2.5 2D and 3D structure modeling and validation

PDBsum (http://www.ebi.ac.uk/thornton-srv/databases/pdbsum/) was used to determine the secondary structure of the new vaccine construct, which was then validated through the PSIPRED server (http://bioinf.cs.ucl.ac.uk/psipred/) (22–24). SWISS Model (https://swissmodel.expasy.org/) was used to model proteins, and then ProSAweb Server (https://prosa.services.came.sbg.ac.at/prosa.php) was used to analyze protein structure and validate the model (25, 26). Godard plot analysis utilizing the ERRAT server (https://servicesn.mbi.ucla.edu/ERRAT/) and RAMPAGE server (http://mordred.bioc.cam.ac.uk/rapper/rampage.php) (27).

### 2.6 Molecular docking with TLRs receptors

Employing TLRs alongside their precisely designed synthetic ligands in vaccines can initiate a potent chain of cytokines, crucial for robust immune reactions (28). Understanding the pattern of interactions among design vaccines with TLR3, TLR4, and TLR8 immune cell receptors is crucial for efficiently inducing immunological responses. The vaccine constructs were docked into the human TLR3 receptor (PDB ID: 2a0z) (29), TLR4 receptor (PDB ID: 4G8A) and TLR8 receptor [PDB ID: 3w3m (29–31)], using web servers Hdock (http://hdock.phys.hust.edu.cn/) and HawkDock (http://cadd.zju.edu.cn/hawkdock/), in order to evaluate the chemical reactions between immune receptors (TLR3, TLR4, and TLR8) and vaccine constructs (V1, V2, V3, and V4) (32). Another webserver HADDOCK (High ambiguity driven protein–protein Docking server) was utilized to generates informative visual representations of the docking outcome, facilitating a comprehensive analysis of the results. These graphical plots allow for easy comparison of the top-docked structure with the complete set of generated structures, providing insights into key parameters such as docking score and RMSD (root mean square deviation) (33).

### 2.7 MD simulation

The best docking results for the SGLV-V4-TLR4 molecule were used to study a chemical dynamics (MD) research simulation. MD simulations, energy efficiency, and protein flexibility were all calculated using the iMODS web server (https://imods.iqfr.csic.es/) (34). iMODS is based on normal mode analysis (NMA) in the internal (dihedral) coordinates of macromolecules that naturally reproduce the collective functional movements of biological macromolecules. Using these modes, iMODS builds pathways for functional transitions between two proteins with homologous structures. The server can simulate potential with several coarse-grained atomic representations and provides an enhanced arrow model based on an affine model to describe the complicated domain dynamics of macromolecules. The service analyses the dynamic molecular structure and the docked protein structure with other ligands as an amino acid of interest in order to deliver elastic network-related data according to NMA, which is equal for the particular instance of deform Eigenvalues, which changes the B-factor (mobility profiles), along with a variation map. The SGLV-V4-TLR4 complex docked PDB file was uploaded to the iMODS service, and results were obtained with all parameters set to their default values (34).

### 2.8 Immune simulation

The C-ImmSim webserver (http://www.cbs.dtu.dk/services/C-ImmSim-10.1/) was used to model computational immunological simulation of our prioritized vaccine design. This platform employs a potent combination of predictive modeling techniques, including Position-Specific Scoring Matrices (PSSM) and a variety of cutting-edge machine-learning algorithms, to assess and predict the cellular and humoral responses elicited by our antigenic vaccine candidate (35). The application leverages antigenic peptide sequences and lymphocyte receptors to replicate the intricate dynamics of immunogenic responses. Throughout our investigation, we precisely observed to a standard clinical protocol, administering two doses of the vaccine with a four-week interval to assess immune responses (36). Our focus lay on six specific human leukocyte antigens: HLA-A0101, HLA-A0201, HLA-B0702, HLA-B3901, HLA-DRB10101, and HLA-DRB10401, each monitored at time intervals of 1, 84, and 168 hours. Immune simulation was executed using the application’s default settings, encompassing 1000 iterative steps (37).

### 2.9 Codon optimization

The JAVA Codon optimization Tool (Jcat) (http://www.jcat.de) was used to optimize the coding and execute a reverse translation of the sequence of amino acid sequences for the suggested immunization (38). After that, an E. coli production gene vector called pET28 was used alongside Snapgene version 5.2 to digitally clone the genetic code sequence (39).

### 2.10 Prediction of the vaccine mRNA secondary structure

The Transcription and Translation web based Tool (http://biomodel.uah.es/en/lab/cybertory/analysis/trans.htm) was employed to acquire the mRNA sequence of the vaccine. To predict the secondary structure of the vaccine mRNA, two web-based servers, Mfold v2.3 (http://www.unafold.org/mfold/applications/rna-folding-form-v2.php) (40) and RNAfold (http://rna.tbi.univie.ac.at/cgi-bin/RNAWebSuite/RNAfold.cgi) (41), were utilized. The primary outcome of interest centered on the minimum free energy (expressed in units of Kcal/mol), with lower values indicating a greater degree of stability within the mRNA’s folding structure.

## 3 Results

### 3.1 Proteins prediction of SGLV vaccine candidate

The complete proteome of the SGLV strain, containing 40 proteins from different strains across the world, was extracted from NCBI in FASTA format (**Supplementary File S1**). Utilizing CD-hit, redundancy was minimized, and human blast yielded four distinct proteins (**Supplementary File S2**). Subsequently, we screened these proteins for allergenicity, antigenicity, and toxicity, identifying optimal candidates with high antigenicity, non-allergen, and non-toxic properties for epitope prediction **(Supplementary Table 1**).

### 3.2 Prediction of T-cell and B-cell and population coverage analysis

For further analysis, Four proteins are selected to recognize the lead epitopes for producing a chimeric vaccine construct against SGLV. T-cell (major histocompatibility complex class I and class II) epitopes were determined for the selected proteins using the IEDB server, with an IC_50_ threshold of 50 nM. The ABCpred scores reached greater than 0.8, and the specificities of the estimated ubiquitous B-cell epitopes were 75%. Vaccines were developed based on predictions of twelve (13) overlapping lead regions for each prioritized protein. the top 12 epitopes based on their antigenicity, IFN positivity, toxicity, and allergenicity (**Table 1**). The main goal was to recognize lead epitopes with the potential to induce humoral and cell-mediated immunogenic responses and host interferons.

**Table 1:** IFN-g epitope prediction, investigation of allergenicity and toxicity, and prediction of overlapping T- and B-cell epitopes.

Protein IDs	MHC-I	IC50	MHC-II	B-cell Epitopes	ABCpred score	IFNepitope score	Allergenicity	Toxicity
YP_010840762.1	CAFGAFPVA	17	SDMVCAFGAFPVAEP	RICSDMVCAFGAFPVA	387	-0.1401593	non-allergen	Non-toxic
	WQDQEIVPV	74	WQDQEIVPVEHMLHQ	SGWQDQEIVPVEHMLH	444	-0.6228511	non-allergen	Non-toxic
	KNKGGTSLA	4.3	GSWTKKNKGGTSLAV	WGSWTKKNKGGTSLAV	215	2	non-allergen	Non-toxic
YP_010840761.1	FAVGPLVPL	10	FAVGPLVPLESAQKV	TKFAVGPLVPLESAQK	988	-0.2917396	non-allergen	Non-toxic
	VAKGLQSSY	42	AIKVEAVAKGLQSSY	EEIQQYLNDCSKGLLN	1261	-0.110257	non-allergen	Non-toxic
	LADSQIETG	28	RNIILADSQIETGTT	SEELLAFVDSQYVLTI	307	-0.0578239	non-allergen	Non-toxic
YP_010840760.1	YGAGQITAL	69	RPSYGAGQITALLDV	GRPSYGAGQITALLDV	1100	-1	non-allergen	Non-toxic
	IAIFHKTPE	13	IAIFHKTPERDLFDL	DIAIFHKTPERDLFDL	963	-0.5001685	non-allergen	Non-toxic
	TALLDVQGL	53	AGQITALLDVQGLLL	GAGQITALLDVQGLLL	1105	-0.1869516	non-allergen	Non-toxic
UWI48350.1	TVKSTESIC	9	EDIKWTVKSTESICE	FEDIKWTVKSTESICE	44	-1	non-allergen	Non-toxic
	VEQTKFAVA	51	ADWVEQTKFAVAPLV	ADWVEQTKFAVAPLVP	4	-0.394556	non-allergen	Non-toxic
	CFEDIKWTV	15	CRYRGCFEDIKWTVK	ECCRYRGCFEDIKWTV	36	-1	non-allergen	Non-toxic

**Figure 3:**
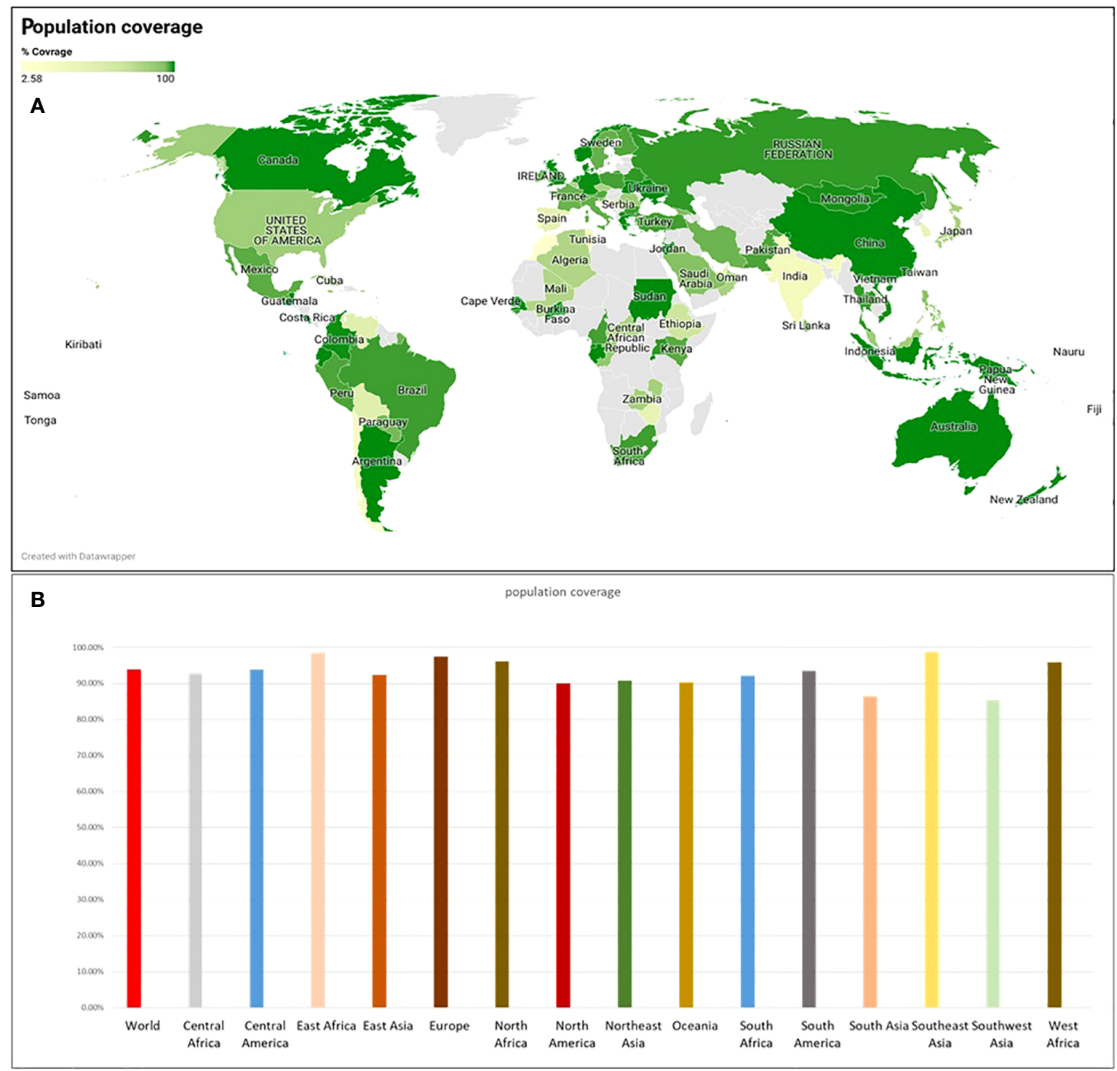
The population coverage was determined using the IEDB webserver, **(A)** population coverage across the world’s countries and **(B)** population coverage across different ethnicities.

The epitopes chosen exhibited 100% coverage across the global population (**Supplementary Table 2**). Analysis from the IEDB database indicated a notably high population coverage for these predicted epitopes, particularly in regions significantly impacted by SGLV, such as east Asia, Europe and south east Asia (**Figure 3**).

### 3.3 Multi-epitope vaccine design

To generate a multi-epitope vaccine EAAAK, CTL, HTL, and GGGS/HEYGAEALERAG linkers and adjuvants were used. When administered intramuscularly, vaccine containing linkers offer superior protection against each epitope (42). Increased immunogenic responses were achieved by coupling the epitopes to various adjuvants, such as HBHA protein molecules, beta-defensin, 50S ribosome enzyme L7/L12 adjuvants, and N-terminally abbreviated HBHA comparable amino acid sequences. Immunization strategies have used the PADRE peptide sequence to protect against difficulties caused by local variations in HLA-DR. Previous studies have shown that vaccine formulations including PADRE provide enhanced immune protection and high cytotoxic T lymphocyte (CTL) responses, as shown in **Supplementary Table 3**.

### 3.4 Immunological and physiochemical properties

Based on their immunological features, none of the immunization strategies were found to be harmful or allergic. Each one of the multi-epitope vaccine formulations has a substantial antigenic property, as demonstrated by antigenicity scores > 0.8 as estimated by the VaxiJen 2.0 server. Using cross-validation on the peptide sequence based on established datasets, VaxiJen 2.0 determines the antigenicity of viral sequences and identifies their protective properties. Each structure VaxiJen 2.0 score fell between 0.4333 and 0.5197, which is the same as the default threshold for viruses (13). Using the ProtParam service, we were able to determine the physiochemical parameters of the vaccine compositions and found that the molecular weights of each epitope in these innovations ranged from 30 kDa to 55 kDa. The selected vaccine designs have GRAVY values around -0.128 and -0.310, indicating that they are hydrophilic. The numerical pI values fell between 8.87 and 10.07. The thermostability of these structures was demonstrated by aliphatic index values between 69.09 and 82.50. The stability of these constructs at different temperatures was projected to be shown by their unpredictability index values, which ranged between 30.93 and 41.81 (**Table 2**). The only real difference among the designs was the adjuvant; therefore, none of them changed much in terms of their physicochemical qualities. The vaccine designs’ ability to elicit robust immunogenic reactions in the human host was deduced from an examination of their immunogenic and physiochemical properties (43). More experimental work is needed to confirm the reliability of these results.

**Table 2:** Physiochemical properties of the vaccine constructs using ProtParam server and JCAT server.

Vaccine constructs	No of Amino Acids	Molecular weight (Da)	Instability index	Theoretical PI	Grand average of hydropathicity (GRAVY )*	GC content	CAI (0.85-1.0)	Aliphatic index
Con#1 adjuvant = HBHA adjuvant	427	43117	41.32 protein as stable	10.07	-0.282	52.22	1.0	69.09
Con#2 adjuvant = Beta definsin adjuvant	512	51396	35.65 protein as stable	9.51	-0.128	51.43	1.0	78.07
Con#3 adjuvant= HBHA conserved adjuvant	541	55585	41.80 protein as unstable	9.55	-0.310	53.23	1.0	75.71
Con#4 adjuvant = Ribosomal protein adjuvant	292	30649	30.93 protein as stable	8.87	0.129	52.73	1.0	82.50

### 3.5 Structures modeling, validation, and refinement

**Figure 4:**
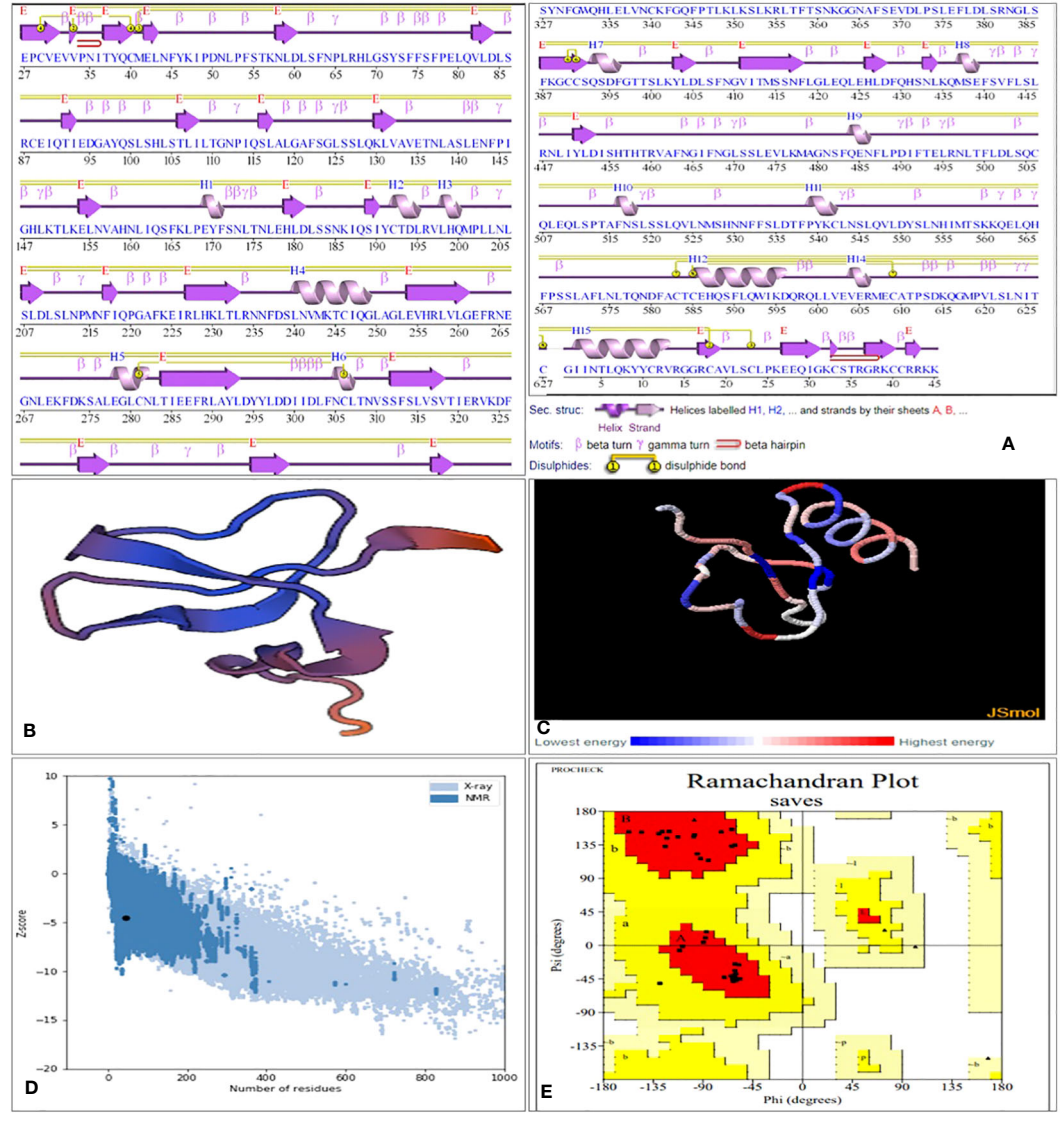
SGLV-V4 three-dimensional structural analysis, refinement, and validation **(A)** Protein’s Secondary Structure with Graceful Elements: strands (elegant pink arrows), helices (royal purple springs), and captivating motifs in shades of red (-hairpins, mesmerizing -turns, and more), **(B)** The Swiss Model designed a 3D model of the multi-epitope vaccination using a homology modeling method. **(C)** Binding energy of the JSmol structure **(D)** ProSA-web yields a Z-score of -4.52. **(E)** Ramachandran plot analysis reveals 90% of the residues, 20% are in the allowed region, and 1% are in the prohibited portion of the plot.

Computational approaches that anticipated secondary structure components were used to analyze the structural characteristics of the vaccine constructs. The PDBsum server displays proteins in their residue conservation and 2D structure. It shows which parts of the protein are not the same (colored blue) and which parts are very similar (colored red). **Figure 4A**. Similarly, the PSIPRED 4.0 server, which uses position-specific scoring matrices (PSSM), was utilized to predict transmembrane helices and topology within the peptide sequence, as well as identify fold and domain regions, as shown in **Supplementary Figure 4**. A stable and functional 3D structure of a vaccine is crucial for studying its molecular interactions with immune receptor proteins. **Figure 4B** displays the vaccine constructions predicted by the homology modeling techniques implemented in the Swiss Modelling server; these constructs were further refined by the DeepRefiner web server and submitted to a physical validation study. The binding energy of the JSmol structure is shown in **Figure 4C**. 73.7% of the V1 construct, 82.8% of the V2, 91.5% of the V3, and 97.4% of the V4 acids remained in the plots’ favorable region (**Figure 4D**), indicating that the vaccine constructions were highly stable. The improved vaccine designs had ERRAT quality ratings between 58% and 97%. The ProSA-web server found that the Z score of all vaccine constructions might range from -0.88 to -4.71 (**Figure 4E**). **Table 3** displays the 3D structural validation of vaccine constructs.

**Table 3:** 3D structural validation of vaccine constructs via ERRAT, PROCHECK (Ramachandran plot favored region), and ProSA-Web Server.

Vaccine Construct	ERRAT (%)	PROCHECK (%)	ProSA (Z-score)
SGLV -V1	58.3333	73.7	-4.71
SGLV -V2	99	82.8	-4.11
SGLV -V3	97.3154	91.5	-0.88
SGLV -V4	93.75	97.4	-4.52

### 3.6 Molecular docking

**Figure 5:**
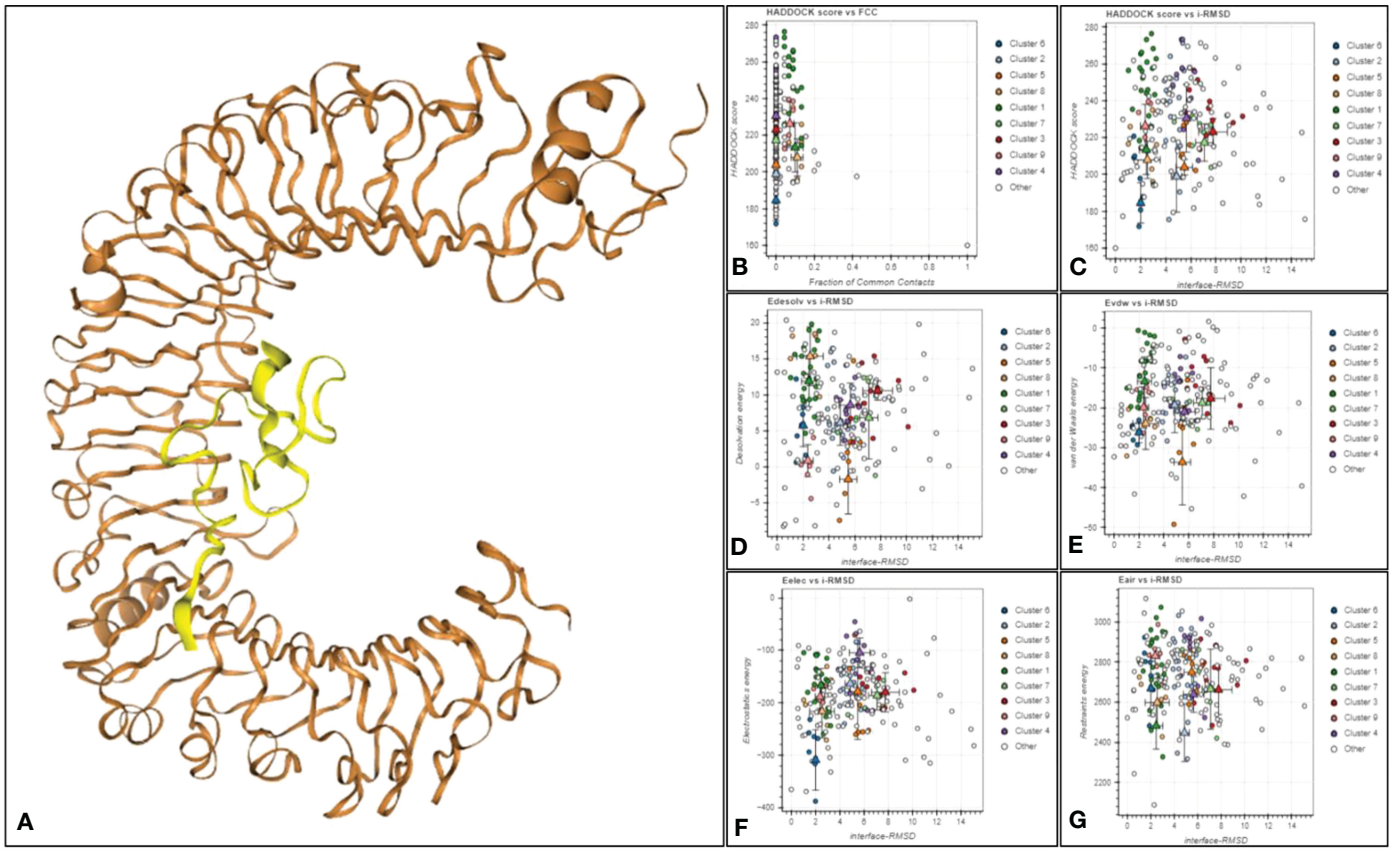
**(A)** The docked complex of TRL4-V4. The TLR4 receptor is depicted in brown, while yellow represents the SGLV-V4 vaccine construct. **(B)** Haddock score against a fraction of frequent contacts. **(C)** Haddock score against ligand RMSD. **(D)** Electrostatic Solvation Energy (EDESOLV) against Initial-RMSD in Molecular Simulations (I-RMSD), **(E)** van der Waals energy against interface I-RMSD, **(F)** Electrostatic energy (Eelec) of docked molecule against interface-RMSD, **(G)** (Ensemble-Averaged Interaction-Reweighted Simulation) EAIR outperforms I-RMSD in predicting the structure of receptor-ligand complexes.

Molecular docking is used for predicting the suitable binding between multi-epitope vaccine (MEV) and receptor molecules. Human surface TLR3, TLR4, and TLR8 immune system receptors were used to dock MEV with the help of Hdock Server (a blind docking technique) and Hawkdock Server. In the result, only one structure is prioritized for each dock based on its high score and lowest binding energy (**Table 4**). And the 3D structure of each docking is shown in **Supplementary Figures 1–3**. In this study, the binding energy of the V4 was found to be lower with TLR4 area by a significant margin (-66.26 kcal/mol) as compared to docking with TLR3 and TLR8 receptors. The prioritized complex TRL4-V4 was then assigned to HADDOCK to evaluate various parameters in our analysis, including HADDOCK scores, cluster size, van der Waals energy, electrostatic energy, desolvation energy, restraints violation energy, buried surface area, and Z score (**Figure 5**). Notably, Cluster 6 exhibited exceptional characteristics with a Z score of -2.0, HADDOCK scores of -9.5, a cluster size of 7, van der Waals energy of -26.2, electrostatic energy of -309.1, desolvation energy of -2.5, restraints violation energy of 2998.6, and a substantial buried surface area of 1708.2. Consequently, we selected the most promising structure from Cluster 6 for molecular dynamics simulation. The docking investigation reveals that the vaccine designs have strong binding capabilities to the TLR4 protein.

**Table 4:** Docking scores and Binding energies of multi-epitope vaccine constructs and TLRs.

Constructs	TLR3 (2a0z)			TLR4 (4G8A)			TLR8 (3w3m)		
	Docking Score		Binding Energy	Docking Score		Binding Energy	Docking score		Binding Energy
	Hdock	Hawkdock		Hdock score	Hawkdock		Hdock score	Hawkdock	
V1	-274.53	-5209.48	-41.79	-298.62	-5097.4	-43.37	-301.63	-4694.39	-43.47
V2	-240.85	-5463.77	-0.26	-200.17	-4634.95	12.82	-240.85	-4098.19	-16.02
V3	-385.59	-4092.04	-0.35	-312.43	-2352.47	-5.81	-368.66	-3037.87	-14.13
V4	-278.24	-4788.78	-35.78	-266.51	-6853.68	-66.26	-274.59	-4318.53	-28.92

### 3.7 Molecular dynamic simulation

**Figure 6:**
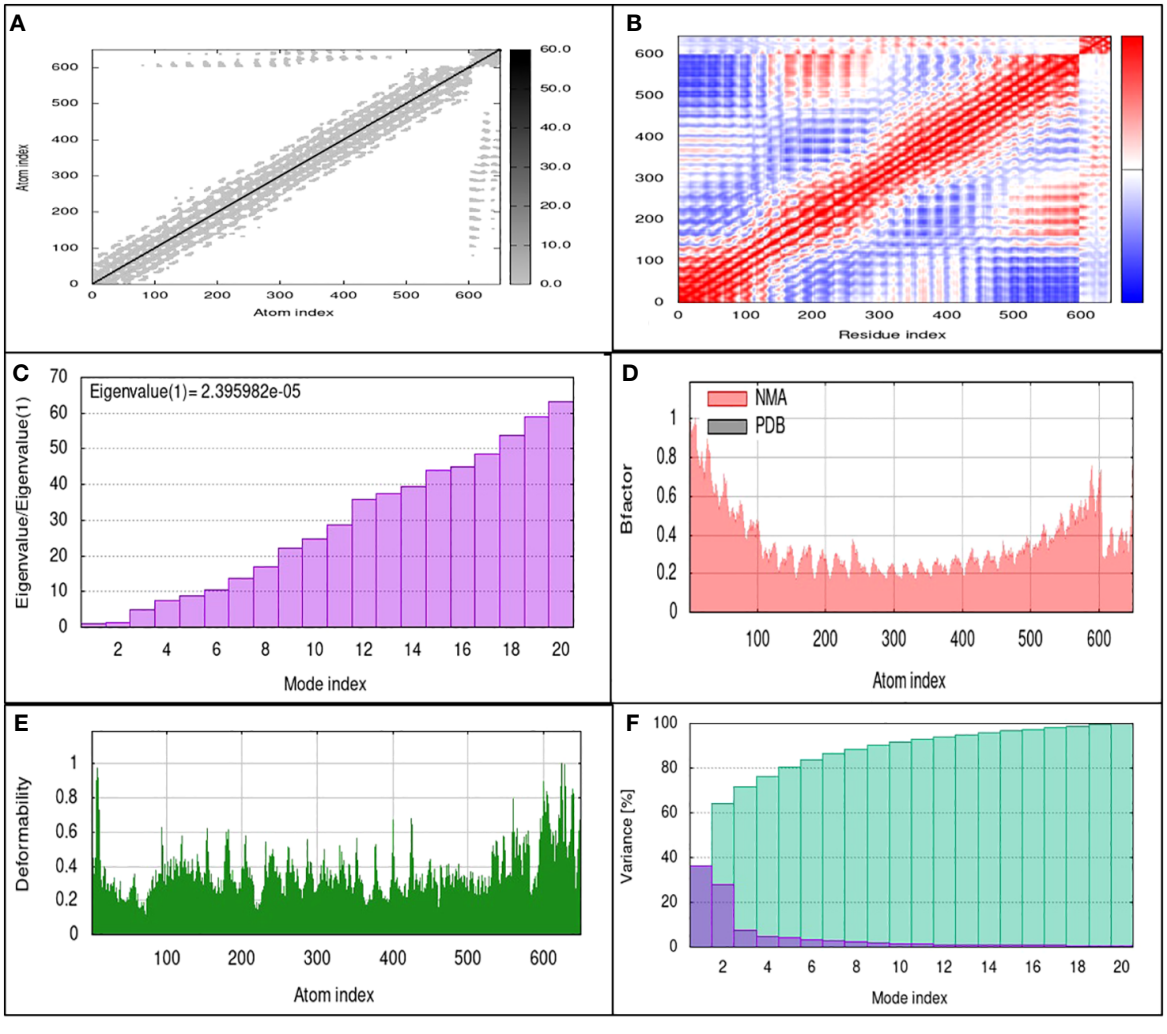
MD simulation results of SGLV-V4 with TLR4 **(A)** The elastic network model uses springs between atoms, indicated by colored dots for stiffness. **(B)** Covariance matrix Shoes paired residue mobility motions, i.e., uncorrelated (white), correlated (red), and anti-correlated (blue). **(C)** Eigenvalues, **(D)** Averaged RMS indicated by B-factor, **(E)** Deformability, and **(F)** Shows variances in Colored bars (purple) represent individuals, and cumulative is represented by green.

The TLR4 receptor was chosen due to its lowest binding energy with the SGLV-V4 construct. To comprehensively evaluate the stability of proteins and the enthalpy efficiency within SGLV-V4-TLR4 complexes, molecular dynamics (MD) simulations were employed. In parallel, the iMODS platform facilitated an in-depth analysis of atomic and molecular movements within the vaccine’s biological context, elucidating macromolecular mobility via the normal mode analysis (NMA) methodology. For a more detailed understanding, **Figure 6** provides a visual representation of the outcomes stemming from MD simulations and NMA conducted on the SGLV-V4 and TLR4 docked complexes. Drawing from the work of Ichiye and Karplus in 1991 (44), we utilized Equation 2 in conjunction with C Cartesian coordinates to compute the correlation matrix, thereby revealing the intricate interplay of atoms through an elastic network model. Each point on the graph symbolizes a spring connecting specific atom pairs, with varying shades of grey denoting differing levels of stiffness (as seen in **Figure 6A**). The complexity of molecular interactions within the system is further elucidated by the covariance map of the complex. By utilizing covariance analysis, this map highlights correlated (red), uncorrelated (white), or anti-correlated (blue) atomic movements, thus providing valuable insights into the dynamics of the complex molecule (**Figure 6B**). Additionally, eigenvalues, reflecting the stiffness of motion, hold a direct proportionality to the energy required for structural deformations. A lower eigenvalue indicates greater ease of deformation for the carbon alpha atoms. Notably, the SGLV-V4-TLR4 complex exhibited an eigenvalue of 2.395982e-05, signifying its stability (as observed in **Figure 6C**).

Furthermore, NMA-derived B-factor analysis was instrumental in portraying the relative amplitude of atomic displacements within the molecular complex. **Figure 6D**, displaying the B-factor graph, illustrates the correlation between the mobility identified in the docked complex NMA and the PDB scores. In this context, RMSD minimization based on local and global structure superposition enabled iterative deformation of the input structure, modeling potential transitions. Meanwhile, the total atomic displacements across all modes of residues at individual atomic sites provide an insightful measure of main-chain deformability. The complex’s deformability graph, illustrated in **Figure 6E**, identifies peak regions representing the protein’s more flexible areas, while inflexible sections exhibit lower values. Additionally, the variance graph, inversely linked to the eigenvalue (as demonstrated in **Figure 6F**), is connected to each normal mode of the complex, elucidating both individual and cumulative variances for a comprehensive depiction of the system’s dynamics.

### 3.8 Immune response simulation

**Figure 7:**
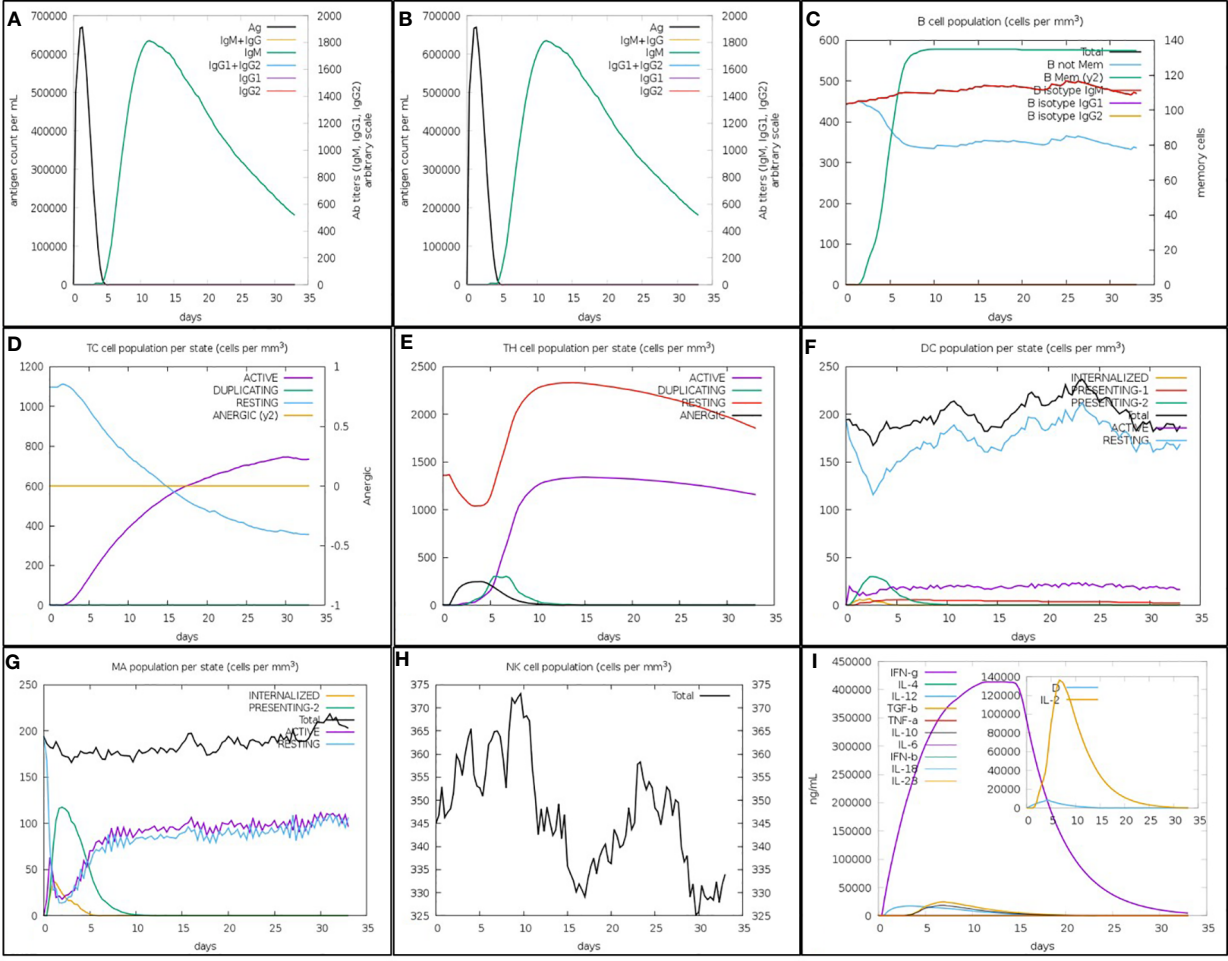
The *in silico* inflamed simulation used by the C-ImmSim Servers allows for an estimation of the SGLV-V4 recombinant peptide vaccination’s immunological potential. **(A)** Vaccines cause an increase in immunoglobin antibodies and a decrease in antigen levels. As seen in **(B)**, B-cell numbers increase and antigen titers fall after immunization. Increased B-cell counts as a result of repeated antigen exposure **(C)**. T-cytotoxic and T-helper cell counts rise **(D, E)** after repeated antigen exposure. Dendritic cells, macrophages, and natural killer cells all grew in number during the vaccination window **(F–H)**. Increased antigen exposure leads to increased cytokine and interleukin **(I)** production. This danger signal is depicted alongside leukocytes and the rate of expansion factor IL-2 in the inset graphic.

The focused MEV significantly boosted secondary responses, as predicted by immune modeling. In principle, this sequence can help the immune system quickly respond to threats. High levels of IgM were the prime simulated response. The simulated secondary and tertiary responses revealed considerable increases in B-cell populations as well as high concentrations of IgG1 + IgG2, IgM, and IgM + IgG antibodies. However, there was a decrease in the antigen levels (**Figures 7A, B**). The increased level of memory B-cell population and isotype switching indicate the formation of immunological memory in this case. Following the subsequent exposure to chimeric antigens, this caused a fast antigen reduction (**Figure 7C**). After further antigen exposure, it was hypothesized that both cytotoxic (TC) and helper (TH) cell subsets would form a similar memory (**Figures 7D, E**). High levels of activity in the macrophage, flare cell, and natural killer cell populations were also sustained during the vaccination period (**Figures 7F–H**). Higher levels of cytokines like interleukins IL-2 and IFN-y were also present (**Figure 7I**). These results provide support for the research showing that the anticipated vaccine formulation induced successful immune reactions against SGLV.

### 3.9 Molecular cloning and codon optimization

**Figure 8:**
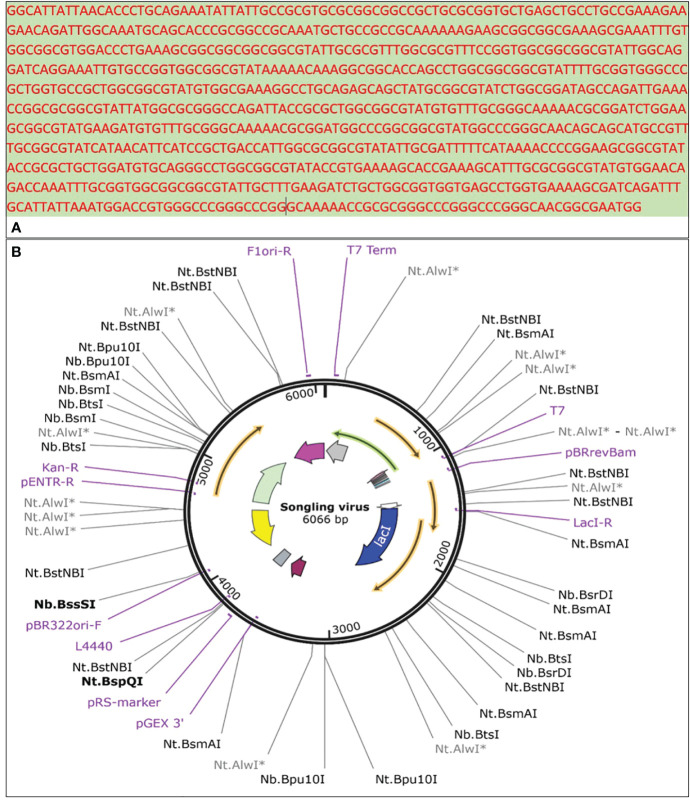
**(A)** Reverse-translated primary DNA sequence of the SGLV-V4, **(B)** SGLV-V4 cloned specifically into the E. coli expression vector [pET28a(+)].

Designing a vaccine with an appropriate expression system is the initial stage in evaluating a vaccination candidate, which requires a serological study. Prior to *in vitro* expression, a similar strategy was employed in earlier experiments for *in silico*-designed vaccines. The bacterial cell expresser E. coli was selected. Cloning and transcription are greatly facilitated by the Java Codon Adaptation Test (JCAT), which makes the E. coli K12 strain a great host organism. The estimated GC content of the improved sequence was 52.7%, which is significantly higher than the value of 50.73 found in E. coli. The modified sequence had a CAI (codon adaptation index) of 1.0. The multi-epitope vaccine (MEV) vector’s codon usage curve is shown in **Figure 8**. Finally, SnapGene software was used to create a recombinant plasmid sequence by inserting the final vaccine construct V4 modified codon sequence into the plasmid vector pET28a (+), ensuring heterologous cloning and expression in the E. coli system (**Figure 8**).

### 3.10 Secondary structure of vaccine mRNA

**Figure 9:**
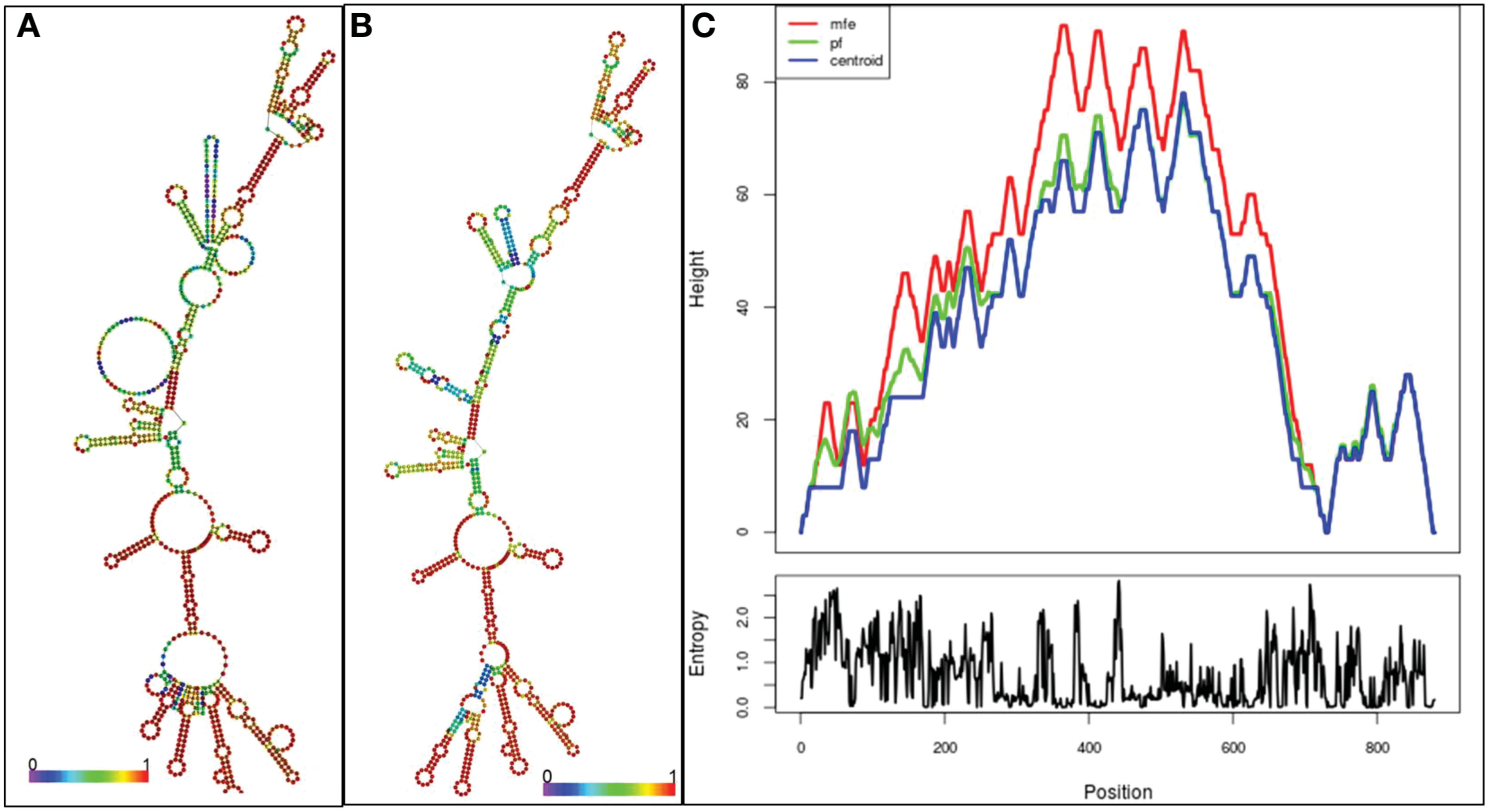
**(A)** Optimal secondary structure of the vaccine mRNA **(B)** Central secondary configuration of the vaccine mRNA **(C)** thermodynamic ensemble of mRNA structure, and the centroid structure are vividly depicted in the mountain plot representation. Additionally, the positional entropy plot unveils the intricacies of each position.

The RNAfold server predicts the vaccine mRNA’s secondary structure with a minimum free energy of -268.90 kcal/mol (**Figure 9A**), while the centroid secondary structure shows -229.37 kcal/mol (**Figure 9B**). mFold v2.3 server calculates the optimal secondary structure’s minimum free energy at -283.12 kcal/mol (**Figure 9C**). A lower minimal free energy suggests greater stability for the vaccine mRNA post-expression *in vivo*.

## 4 Discussion

In response to the growing concern over the flow of Songling virus (SGLV) cases worldwide and the absence of available vaccines, this study investigated the challenge of preventing future Songling virus epidemics. Employing cutting-edge immunoinformatics techniques, we embarked on designing innovative multi-epitope Songling virus vaccine constructs by examining the proteome of the Songling virus to pinpoint targets for a potential vaccine. Using strict standards, they forecasted epitopes for B-cells, MHC-I, and MHC-II. These epitopes play a crucial role by sparking a protective immune response that blocks viruses and establishes long-term defense (45).

The overlapped epitopes are prioritized from MHCI & II, and B-cell which are highly antigenic, non-allergen, and produce humoral response, that combats infections by eliminating infected cells or releasing antiviral substances to establish lasting immunity (46). To enhance this response, a novel vaccine was created using various CTL and HTL segments combined with specialized suitable linkers and adjuvants. Additionally, the vaccine’s design incorporates EAAAK, AAY, and GPGPG linkers and adjuvants, which improve the structure and stability of the vaccine. Four vaccine constructs were designed from selected epitopes. These vaccine models displayed impressive traits: high antigenicity, non-allergenicity, and non-toxicity. Analysis of the vaccine’s physiochemical characteristics indicated its robustness, alkaline nature, and hydrophobic properties, all of which indicate its potential to induce potent and targeted immunogenic responses in infected individuals.

Molecular docking analysis was then employed to explore the interaction between the vaccine constructs and the crucial immune cell receptors, i.e., TLRs. TLRs are known for their pivotal role in immune cell activation and the recognition of viral peptide structures (46). The results revealed strong binding affinities of SGLV-V4 toward TLR4, suggesting that the designed vaccine constructs have the capacity to generate robust immunogenic responses upon exposure. The C-ImmSim server, an immune response evaluation tool, was used to assess a newly designed vaccine’s ability to induce an immunological response. This method simulates key components of the mammalian immune system and tracks how various immune cells respond to the vaccine (7). The goal is to design a vaccine that not only offers immediate protection but also triggers a long-lasting immune response, simulating natural immunity. The top-ranked vaccine candidate, SGLV-V4, activated essential immune components, including antibodies (IgG and IgM), T-cells, B-cells, and cytokines (**Figure 7**) (47). This multi-epitope-based subunit vaccine shows suitable in protecting against the Songling virus. For further investigation to assess the stability and biomolecular process of SGLV-V4, the molecular dynamic simulation and NMA were performed. To enhance vaccine expression, computational cloning was performed on a pET28a (+) vector after codon optimization with the JCAT web service. The optimized codon adaptation index (CAI) and GC content fall within acceptable ranges, ensuring efficient expression in E. coli (strain K12). This optimization is crucial for successful vaccine production.

As we progress toward the next critical steps, we anticipate *in vitro* immunological assays to be conducted to confirm and validate the immunogenicity of the designed vaccine. Subsequently, a challenge-protection preclinical trial will be initiated, presenting a crucial opportunity to rigorously evaluate and substantiate the efficacy and safety of the SGLV-V4-TLR4 vaccine construct. These endeavors aim to provide a comprehensive framework to combat Songling virus infections effectively, potentially mitigating their impact and safeguarding public health against this evolving threat. However, to determine the vaccine’s safety and efficacy, further experimental validation is required, which may involve the production of vaccine proteins with thorough *in vivo* and *in vitro* tests. However, the current research relies entirely on the results of computational approaches for technical equipment.

## 5 Conclusion

In our study on Songling virus (SGLV), a tick-borne pathogen lacking treatment or vaccines, we employed immunoinformatics to identify four potential vaccine target proteins. Designing a comprehensive vaccine candidate with broad global coverage, we combined B-cell and T-cell epitopes and validated them through IFN-γ epitopes. SGLV-V4, selected for its strong performance in molecular docking and favorable properties, was efficiently expressed in E. coli bacteria. Immunological research and simulations confirmed its stability and robust immune response, offering a promising avenue for safer and more effective SGLV-V4 vaccine development.

## Data availability statement

The original contributions presented in the study are included in the article/**Supplementary Material**. Further inquiries can be directed to the corresponding author.

## Author contributions

SA: Writing – original draft, Writing – review & editing, Conceptualization, Data curation, Formal Analysis, Investigation, Methodology. AAli: Writing – original draft, Writing – review & editing, Investigation, Software, Visualization. AAla: Funding acquisition, Validation, Writing – review & editing. AB: Validation, Writing – review & editing. MD: Writing – review & editing. AAb: Writing – review & editing.
