# Supplementary material for: Genomic annotation for vaccine target identification and immunoinformatics-guided multi-epitope-based vaccine design against Songling virus through screening its whole genome encoded proteins
Source: Front Immunol. 2023 Nov 28;14:1284366. doi: 10.3389/fimmu.2023.1284366 (PMC10715409; doi:10.3389/fimmu.2023.1284366)
Supplement: Supplementary file 1 [file DataSheet_1.docx]

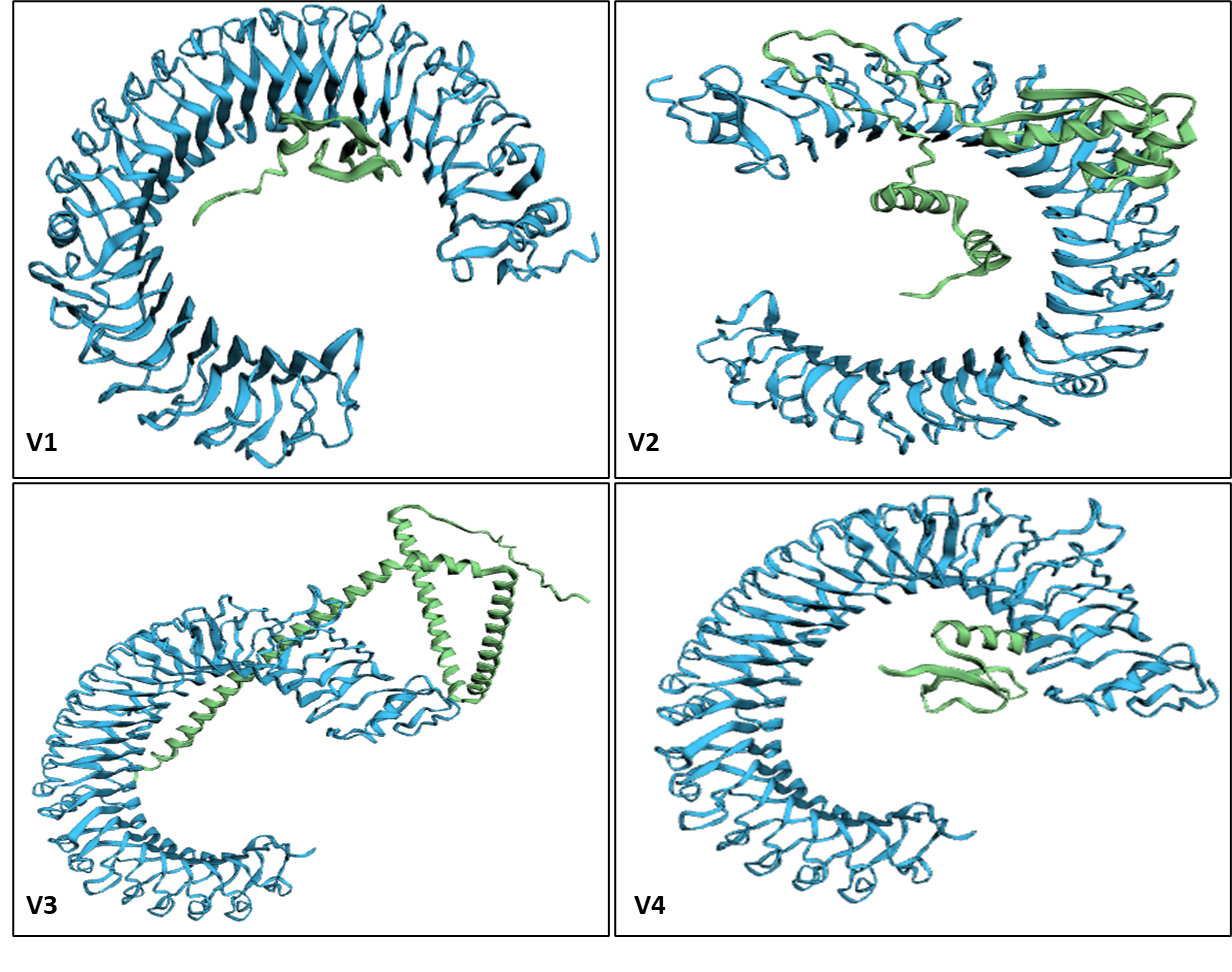


Figure S1: Molecular Docking of TLR3 with multi-epitope vaccine designs, Green shows vaccine construct and blue illustrate the receptors.


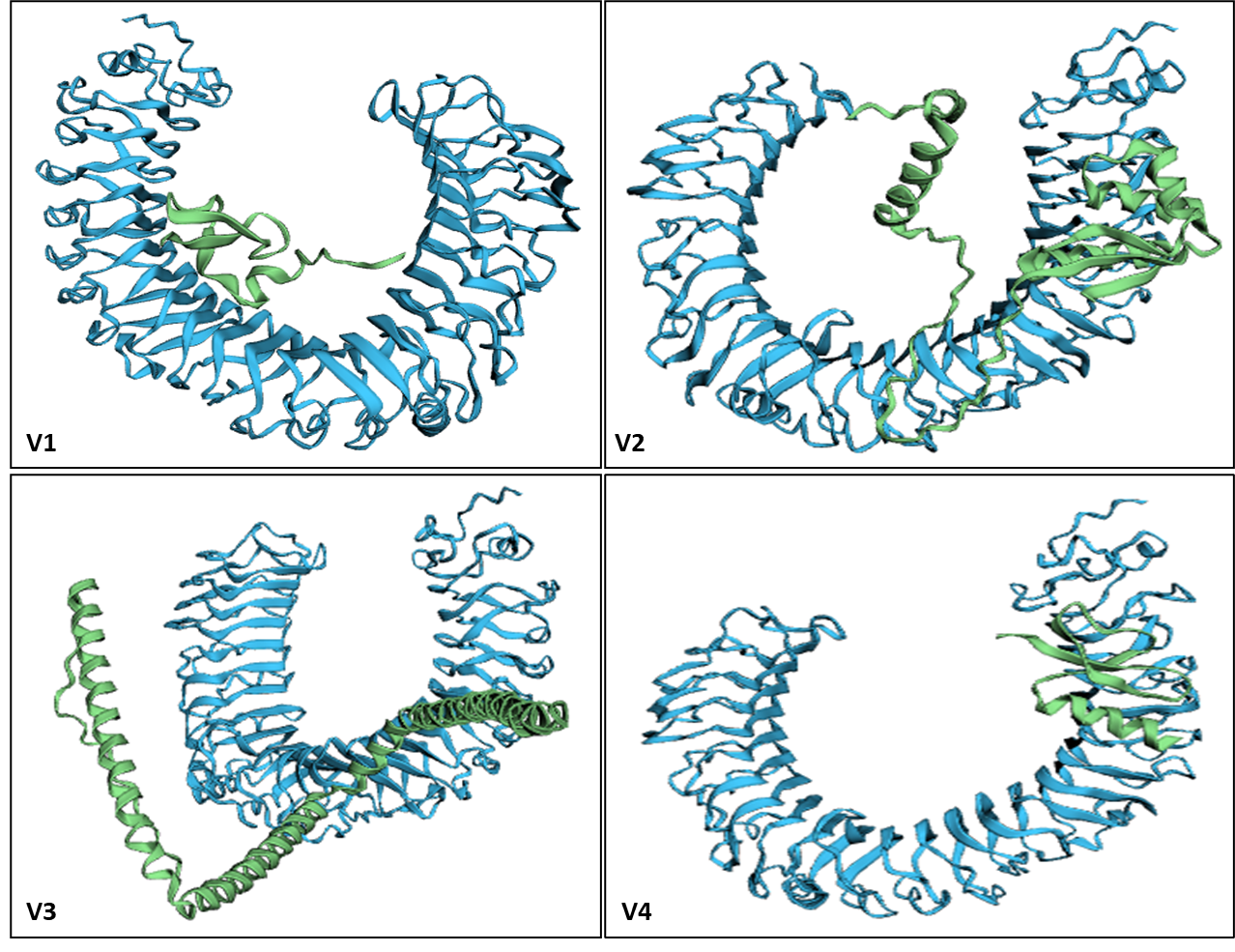


Figure S2: Molecular Docking of TLR4 with multi-epitope vaccine designs, Green shows vaccine construct and blue represents the receptors.


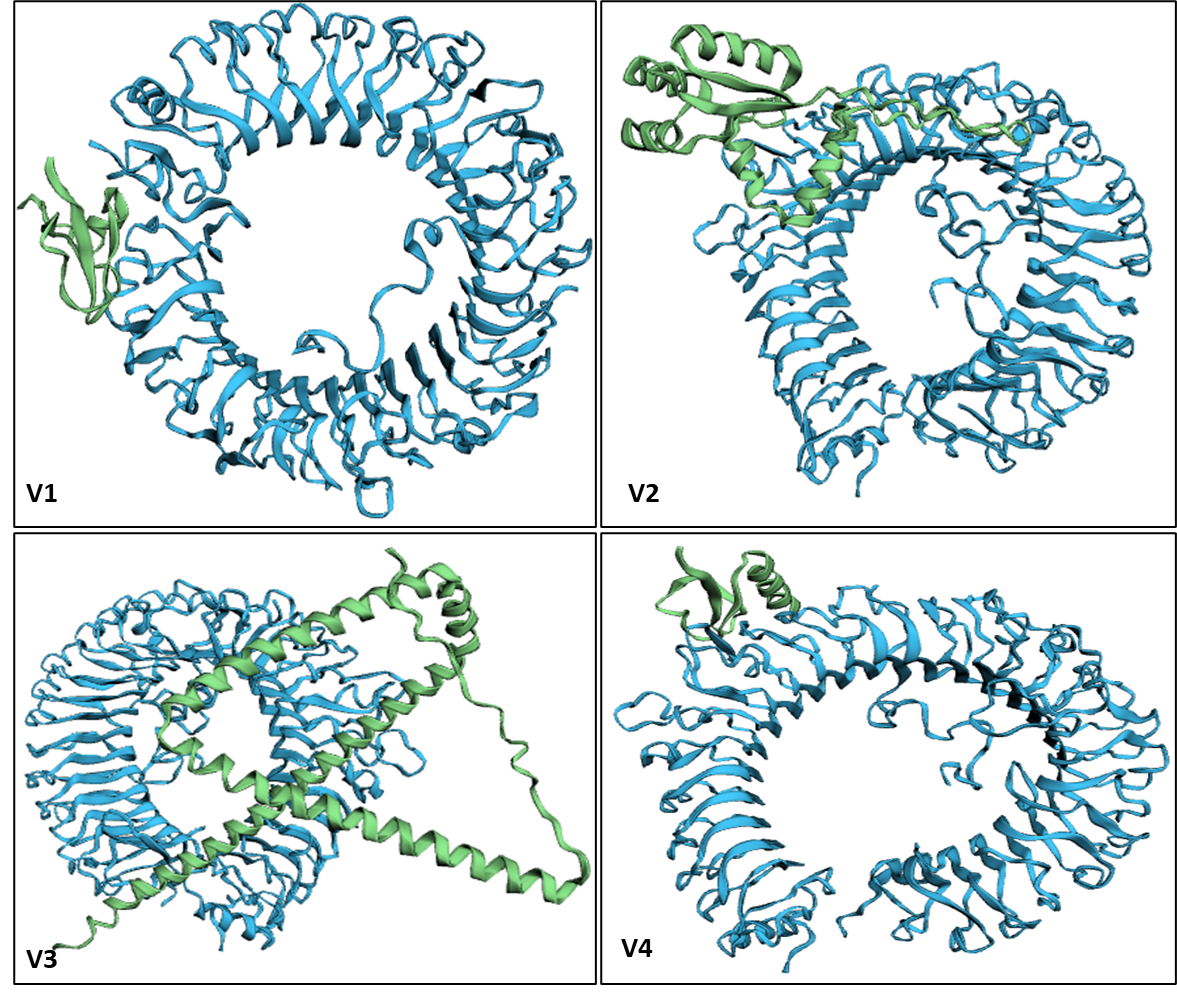


Figure S3: Molecular Docking of TLR8 with multi-epitope vaccine designs, Green shows vaccine construct and blue shows the receptors.


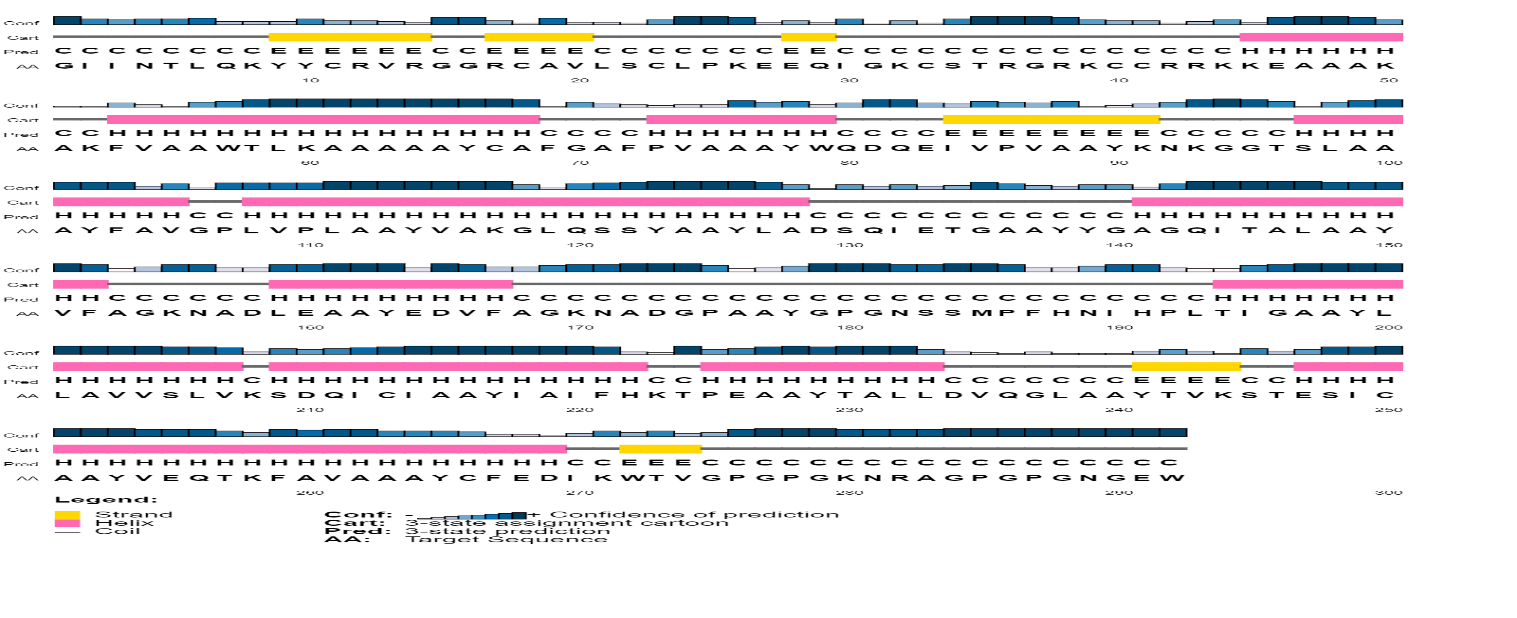


Figure S4: secondary structure predication of vaccine construct by PSIPRED webserver.

Table 1. The top-ranked proteins for vaccine candidate of SGLV for designing MEV constructs models.

| **Proteins Ids** | **Protien name** | **Allergenicity** | **Toxicity** | **Antigenicity** |
| --- | --- | --- | --- | --- |
| YP_010840762.1 | Nucleocapsid protein | Non-allergen | Non-toxic | 0.4392 |
| YP_010840761.1 | RNA-dependent RNA polymerase | Non-allergen | Non-toxic | 0.4334 |
| YP_010840760.1 | glycoprotein precursor | Non-allergen | Non-toxic | 0.5197 |
| UWI48350.1 | RNA-dependent RNA polymerase, partial | Non-allergen | Non-toxic | 0.4898 |

**Table S2.** Population Coverage of MHC Across Different Ethnicities

|  | YP_010840762.1 | | YP_010840761.1 | | YP_010840760.1 | | UWI48350.1 | |
| --- | --- | --- | --- | --- | --- | --- | --- | --- |
| Region | MHC1 | MHC2 | MHC1 | MHC2 | MHC1 | MHC2 | MHC1 | MHC2 |
| World | 97.99% | 92.84% | 98.00% | 99.26% | 98.39% | 99.40% | 95.67% | 97.79% |
| Central Africa | 9.07% | 83.91% | 9.07% | 58.10% | 97.07% | 58.10% | 6.26% | 34.25% |
| Central America | 98.39% | 82.41% | 98.39% | 99.49% | 98.74% | 99.60% | 97.19% | 98.69% |
| East Africa | 99.64% | 87.47% | 99.75% | 99.96% | 100.00% | 100.00% | 99.79% | 99.94% |
| East Asia | 99.99% | 96.07% | 99.99% | 100.00% | 100.00% | 100.00% | 99.99% | 100.00% |
| Europe | 99.59% | 90.03% | 99.60% | 99.90% | 99.60% | 99.90% | 98.79% | 99.54% |
| North Africa | 99.91% | 60.80% | 99.93% | 99.99% | 100.00% | 10000% | 99.95% | 99.99% |
| North America | 97.95% | 60.21% | 98.55% | 99.43% | 98.55% | 99.43% | 97.03% | 98.55% |
| Northeast Asia | 99.55% | 32.10% | 99.15% | 99.66% | 99.15% | 99.66% | 98.51% | 99.27% |
| Oceania | 92.91% | 63.52% | 99.55% | 99.69% | 99.64% | 99.75% | 99.33% | 99.38% |
| South Africa | 98.91% | 76.44% | 97.70% | 99.16% | 100.00% | 100.00% | 98.96% | 99.40% |
| South America | 98.31% | 88.83% | 98.92% | 99.75% | 100.00% | 100.00% | 99.81% | 99.93% |
| South Asia | 98.63% | 85.29% | 98.93% | 99.56% | 100.00% | 100.00% | 99.20% | 99.60% |
| Southeast Asia | 99.07% | 95.81% | 98.64% | 99.25% | 98.66% | 99.27% | 96.90% | 98.05% |
| Southwest Asia | 99.54% | 90.02% | 99.07% | 99.68% | 99.12% | 99.70% | 97.31% | 98.84% |
| West Africa | 99.88% | 93.81% | 99.89% | 99.98% | 99.57% | 99.87% | 98.92% | 99.58% |
| West Indies | 93.09 | 96.85% | 99.79% | 99.88% | 100.00% | 100.00% | 99.93% | 99.98% |
| Average | 97.99% | 79.97%. | 93.45 | 97.05 | 99.23 | 97.33 | 93.15 | 95.46 |

Table S3: multi-epitope vaccine constructs.

| **Vaccine constructs** | **No of Amino Acids** | **Primary structure of vaccine constructs** |
| --- | --- | --- |
| Con#1 adjuvant = HBHA adjuvant | 427 | EAAAKGIINTLQKYYCRVRGGRCAVLSCLPKEEQIGKCSTRGRKCCRRKKEAAAKAKFVA  AWTLKAAAGGGSCAFGAFPVAGGGSWQDQEIVPVGGGSKNKGGTSLAGGGSFAVGPLVP  LGGGSVAKGLQSSYGGGSLADSQIETGGGGSYGAGQITALGGGSVFAGKNADLEGGGSED  VFAGKNADGPGGGSGPGNSSMPFHNIHPLTIGGPGPGLLAVVSLVKSDQICIGPGPGIAIFHK  TPEGPGPGTALLDVQGLGPGPGTVKSTESICGPGPGVEQTKFAVAGGGSCFEDIKWTVGPG  PGGALASCMGLIYNRMGKKPLRERRRKKPLTIGEKKLVLATGLRNSPLRERRRKRKKGENS  WLGKKGFAFLDKKSWLGRTKKVFAGKNAKKVFAGKNKKGKNADLKKAKFVAAWTLKAA  AGGGS |
| Con#2 adjuvant = Beta definsin adjuvant | 512 | EAAAKMAKLSTDELLDAFKEMTLLELSDFVKKFEETFEVTAAAPVAVAAAGAAPAGAAVE  AAEEQSEFDVILEAAGDKKIGVIKVVREIVSGLGLKEAKDLVDGAPKPLLEKVAKEAADEA  KAKLEAAGATVTVKEAAAKAKFVAAWTLKAAAGGGSCAFGAFPVAGGGSWQDQEIVPVG  GGSKNKGGTSLAGGGSFAVGPLVPLGGGSVAKGLQSSYGGGSLADSQIETGGGGSYGAGQI  TALGGGSVFAGKNADLEGGGSEDVFAGKNADGPGGGSGPGNSSMPFHNIHPLTIGGPGPGLL  AVVSLVKSDQICIGPGPGIAIFHKTPEGPGPGTALLDVQGLGPGPGTVKSTESICGPGPGVEQTK  FAVAGGGSCFEDIKWTVGPGPGGALASCMGLIYNRMGKKPLRERRRKKPLTIGEKKLVLATG  LRNSPLRERRRKRKKGENSWLGKKGFAFLDKKSWLGRTKKVFAGKNAKKVFAGKNKKGKN  ADLKKAKFVAAWTLKAAAGGGS |
| Con#3 adjuvant= HBHA conserved adjuvant | 541 | EAAAKMAENPNIDDLPAPLLAALGAADLALATVNDLIANLRERAEETRAETRTRVEERRARLTKFQEDLP  EQFIELRDKFTTEELRKAAEGYLEAATNRYNELVERGEAALQRLRSQTAFEDASARAEGYVDQAVELTQE  ALGTVASQTRAVGERAAKLVGIELEAAAKAKFVAAWTLKAAAGGGSCAFGAFPVAGGGSWQDQEIVPV  GGGSKNKGGTSLAGGGSFAVGPLVPLGGGSVAKGLQSSYGGGSLADSQIETGGGGSYGAGQITALGGG  SVFAGKNADLEGGGSEDVFAGKNADGPGGGSGPGNSSMPFHNIHPLTIGGPGPGLLAVVSLVKSDQIC  IGPGPGIAIFHKTPEGPGPGTALLDVQGLGPGPGTVKSTESICGPGPGVEQTKFAVAGGGSCFEDIKWTV  GPGPGGALASCMGLIYNRMGKKPLRERRRKKPLTIGEKKLVLATGLRNSPLRERRRKRKKGENSWLGKKG  FAFLDKKSWLGRTKKVFAGKNAKKVFAGKNKKGKNADLKKAKFVAAWTLKAAAGGGS |
| Con#4 adjuvant = Ribosomal protein adjuvant | 292 | GIINTLQKYYCRVRGGRCAVLSCLPKEEQIGKCSTRGRKCCRRKKEAAAKAKFVAAWTLKAAA  AAYCAFGAFPVAAAYWQDQEIVPVAAYKNKGGTSLAAAYFAVGPLVPLAAYVAKGLQSSYA  AYLADSQIETGAAYYGAGQITALAAYVFAGKNADLEAAYEDVFAGKNADGPAAYGPGNSSM  PFAAYHNIHPLTIGAAYIAIFHKTPEAAYTALLDVQGLAAYTVKSTESICAAYVEQTKFAVAAAY  CFEDLLAVVSLVKSDQICIIKWTVGPGPGKNRAGPGPGNGEW |
